# Determination of thyroid volume by ultrasound: a valuable tool for the investigation of congenital hypothyroidism

**DOI:** 10.1590/0100-3984.2021.54.3e2

**Published:** 2021

**Authors:** Maria Cristina Chammas

**Affiliations:** 1 Director of the Ultrasound Department at the Hospital das Clínicas da Faculdade de Medicina da Universidade de São Paulo (HC-FMUSP), Professor in the Graduate Program in Radiology and Oncology at the FMUSP, and Coordinator of Ultrasound Services for the DASA Group, São Paulo, SP, Brazil; President-Elect of the World Federation for Ultrasound in Medicine and Biology (2019-2021). Email: cristina.chammas@hc.fm.usp.br.

Congenital hypothyroidism is a neonatal disease characterized by low levels of thyroid hormones. The most common causes of primary congenital hypothyroidism are dysgenesis and dyshormonogenesis, which are defined as defects in the development of the thyroid gland and as defects in the synthesis of thyroid hormones, respectively^(1-3)^.

All neonates should undergo screening for congenital hypothyroidism, which involves determination of the levels of thyroid-stimulating hormone, T4, or both^(1-3)^. When those levels are abnormal, the patient is referred for clinical evaluation and periodic follow-up examinations. The methods currently employed to establish the cause of congenital hypothyroidism include ultrasound of the thyroid and radionuclide scintigraphy, the latter with either ^123^I or ^99m^Tc-pertechnetate^(4,5)^.

Ultrasound of the thyroid is a rapid method that, unlike scintigraphy, does not require specific preparation. Consequently, ultrasound can be performed as an initial exam and at any time^(1)^. Ultrasound has several advantages and is the main diagnostic imaging tool for thyroid studies^(1)^, because it can detect abnormalities of the thyroid gland, either in terms of volume, morphology, echogenicity or echotexture of its parenchyma, as well as being able to identify focal or diffuse lesions.

In cases of congenital hypothyroidism, ultrasound plays a determining role in the diagnosis. In patients with a normal or enlarged thyroid gland and low or undetectable levels of thyroglobulin, the etiology is thyroglobulin deficiency; in such patients, it is redundant to perform scintigraphy and intravenous perchlorate discharge testing^(1)^. However, patients with a normal or enlarged thyroid gland and normal or high levels of thyroglobulin should undergo scintigraphy to identify defects in organogenesis or the sodium/iodide symporter, which is an integral plasma membrane glycoprotein that mediates the active transport of iodine to thyroid follicular cells, which is the first step in thyroid hormone biosynthesis. Therefore, in cases under investigation for congenital hypothyroidism, it is extremely important to establish the thyroid volume and to know the range of normality. Unfortunately, there are few reference values for thyroid volume, as calculated by ultrasound, in euthyroid children under 6 years of age^(6,7)^. The reference values that do exist may not correspond to the reality in Brazil, given that thyroid volume can vary not only with height but also with iodine intake, sex, body surface area, and the stage of puberty^(1)^.

In view of the above, the article authored by Souza et al.^(8)^, which was published in the previous issue of **Radiologia Brasileira**, is extremely relevant for the field of pediatric ultrasound in Brazil. The authors determined the mean thyroid volume for each age group, between 5 days and 3 years of age, among children in southeastern Brazil, which is currently considered an iodine-sufficient region, as is the case for most regions of Brazil. Souza et al.^(8)^ reported mean thyroid volumes and established reference values, regardless of sex and height, for two age groups: below 2 months; and between 2 months and 3 years. Although it was a preliminary study, with a small sample, the enormous contribution of the work is that is provides reference values for thyroid volume in euthyroid children, in an age range not previously addressed, within the context of the reality of Brazil. Certainly, the reading of the article will be worthwhile for those who work in the field of pediatric ultrasound and the values obtained by the authors will be adopted as references.
